# On-Line Organic Solvent Field Enhanced Sample Injection in Capillary Zone Electrophoresis for Analysis of Quetiapine in Beagle Dog Plasma

**DOI:** 10.3390/molecules21010121

**Published:** 2016-01-21

**Authors:** Yuqing Cao, Jun Wen, Tingting Zhou, Guorong Fan

**Affiliations:** 1 Department of Pharmaceutical Analysis, School of Pharmacy, Second Military Medical University, Shanghai 200433, China; caoyuqing1109@163.com (Y.C.); wenjunapple@163.com (J.W.); 2 Shanghai Key Laboratory for Pharmaceutical Metabolite Research, Shanghai 200433, China; 3 Department of Clinical Pharmacy, Shanghai General Hospital, School of Medicine, Shanghai Jiaotong University, No. 100 Haining Road, Shanghai 200025, China

**Keywords:** quetiapine fumarate, capillary zone electrophoresis, field enhanced sample injection

## Abstract

A rapid and sensitive capillary zone electrophoresis (CZE) method with field enhanced sample injection (FESI) was developed and validated for the determination of quetiapine fumarate in beagle dog plasma, with a sample pretreatment by LLE in 96-well deep format plate. The optimum separation was carried out in an uncoated 31.2 cm × 75 μm fused-silica capillary with an applied voltage of 13 kV. The electrophoretic analysis was performed by 50 mM phosphate at pH 2.5. The detection wavelength was 210 nm. Under these optimized conditions, FESI with acetonitrile enhanced the sensitivity of quetiapine about 40–50 folds in total. The method was suitably validated with respect to stability, specificity, linearity, lower limit of quantitation, accuracy, precision and extraction recovery. Using mirtazapine as an internal standard (100 ng/mL), the response of quetiapine was linear over the range of 1–1000 ng/mL. The lower limit of quantification was 1 ng/mL. The intra- and inter-day precisions for the assay were within 4.8% and 12.7%, respectively. The method represents the first application of FESI-CZE to the analysis of quetiapine fumarate in beagle dog plasma after oral administration.

## 1. Introduction

**Figure 1:**
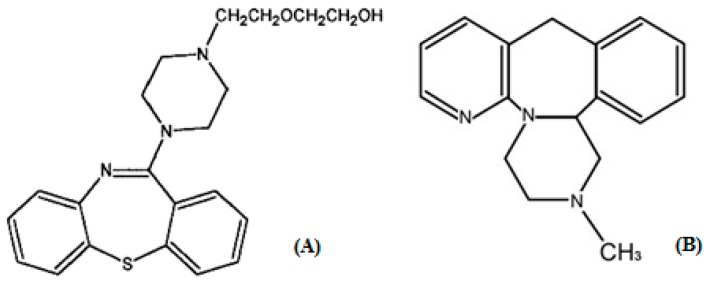
Chemical structures of quetiapine (**A**) and internal standard mirtazapine (**B**).

Quetiapine fumarate (QTP), whose chemical name is bis[2-(2-[4-(dibenzo[*b*,*f*][1,4]thiazepin-11-yl)]ethoxy)ethanol]fumarate, Figure 1A, is a dibenzothiazepine derivative with an atypical neuropharmacological profile. Studies [1,2,3,4] have showed that quetiapine is very effective for negative and positive schizophrenia, as well as cognitive impairment, with slightly choline resistant and rarely granulocytopenia. Due to the highest serotonin/dopamine binding ratio, quetiapine makes the serotonin type 2 (5-HT_2_)-receptor blocking effect about twice as strong as the dopamine D_2_-receptor blocking effect. As a result of this binding pattern, the extrapyramidal side effects of quetiapine are minimal [3,5,6,7,8]. Those characteristics make quetiapine well tolerated and effective in patients who have Alzheimer’s disease or Parkinson’s disease [3,4]. In addition, quetiapine is metabolized by the CYP450 system, primarily by CYP3A4 enzyme [5,9].

To the best of our knowledge, many methods had been described so far in the literature for the determination of quetiapine, including high-performance liquid chromatography with ultraviolet detection (HPLC-UV) [10,11,12], UPLC-UV [13], and liquid chromatography coupled with tandem mass spectrometry (LC-MS/MS) [14,15,16]. The detection limit of UV generally can only reach μg level. HPLC-MS/MS, which is expensive for most laboratories, has been the most common choice for this purpose to date. While the obvious advantages of CE are simplicity, economies, high efficiency, good selectivity, small sample volume, and short analysis time. Moreover, various separation modes can be selected for different analytes. Vincenzo Pucci *et al.* [17] used a spectrophotometric and CZE method to determinate quetiapine in commercial tablets for the quality control. However, when we used ultraviolet detection, the sensitivity of CE was always limited because of the little injection volume and small detection window. To resolve this problem, some stacking CE methods were developed to enhance sensitivity [18,19,20,21], such as field amplified sample stacking (FASS), field enhanced/amplified sample injection (FESI), dynamic pH junction or large volume sample stacking (LVSS), and using ionic liquid during pretreatment. In addition, FESI was the easiest sample stacking method by electrokinetic injection that the conductivity of the BGE was at least ten folds than sample due to the simple requirement. However, there was no literature about the quantitative analysis of quetiapine using any stacking CE method until now.

Therefore, the aim of this paper was to develop and validate a simple and rapid CZE method for the determination of quetiapine in beagle dog plasma. For this purpose, a new FESI-CZE method was developed, optimized and validated in terms of precision, linearity, accuracy, robustness, and detection and quantitation limits for determination of quetiapine.

## 2. Results and Discussion

### 2.1. Method Development

In this study, we explored the optimization and validation of preconcentration process and separation mode. Both quetiapine and IS had strong UV absorption at 210 nm. For this reason, the detection wavelength was set at 210 nm.

#### 2.1.1. Optimization of Buffer Specie and Buffer Concentration and pH and Voltage

**Figure 2:**
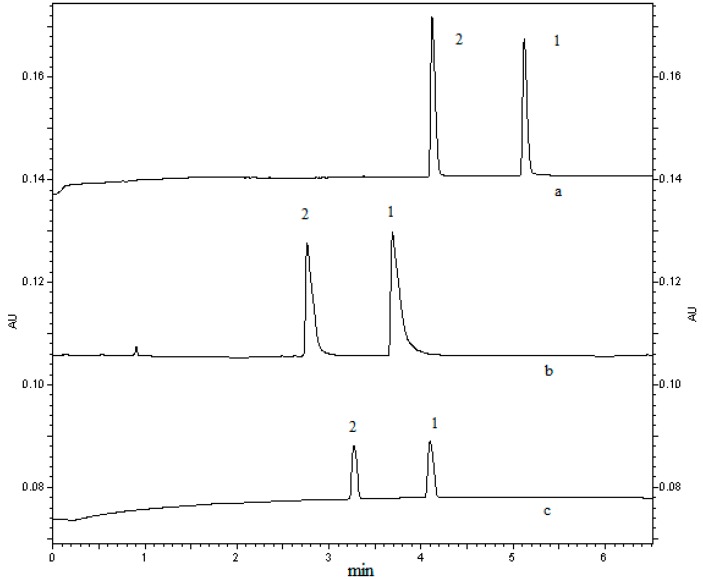
The effect of different buffers on the peak shape of quetiapine fumarate (1000 ng/mL) standard and IS (1000 ng/mL). Capillary, 31.2 cm × 75 μm i.d., effective length 21 cm, uncoated; sample solvents, distilled water; electrokinetic injection, 7.5 kV × 10 s; applied voltage, 10 kV(+)→(−); column temperature, 25 °C; detection 210 nm. Electropherograms: (a) acetic acid-sodium acetate; (b) formic acid-sodium formate; (c) phosphoric acid-sodium phosphate. Peak 1: quetiapine fumarate; peak 2: IS.

Because different buffer solutions have different influence on the EOF and the current produced in the capillary, the effects on the separation performance and peak shape was various [22]. At the beginning of this experiment, three different buffers (acetic acid-sodium acetate, formic acid-sodium formate, phosphoric acid-sodium phosphate) were selected to be studied. As was shown in Figure 2, the peak shapes of analytes were pretty good when we used phosphate as electrolyte at this experiment. In CE analysis, the concentration of buffer is very important. If the concentration of electrolyte is too high, the current will be increased and the EOF will be out of shape, leading to more joule heating and longer analysis time. While lower concentration of buffer will decrease the sensitivity of method because of the lower ionic strength. Considering of the effects of electrolyte’s concentration and the demand of peak shape, 50 mM phosphate buffer was selected.

Based on the pKa value (6.8) of quetiapine, measurements were carried out at lower pH values, which can make quetiapine easily charged, in order to acquire a proper migration time and good resolution of quetiapine and IS. Running buffer contained 50 mM phosphate was studied at different pH (2.0–5.0) to observe the influence on electrophoretic behavior of quetiapine and IS. Better resolution and analysis time were acquired when pH < 3.0, so a running buffer contained 50 mM phosphate with a pH of 2.5 was selected.

Different separation voltages were also tested, such as 11, 12, 13, 14, and 15 kV. While too large separation voltage could cause certain current problems, including high joule heating and low column efficiency. A high column efficiency and short separation time could be obtained when 13 kV was chosen in the experiments.

#### 2.1.2. Selection of Injection Mode and Sample Solvent

Hydrodynamic injection and electrokinetic injection are two injection modes frequently used in capillary electrophoresis. By the hydrodynamic injection, all components including many endogenous interferences can be easily introduced to the capillary, resulting in some interferences when conducting bio-sample analysis. However, the electrokinetic injection mode could avoid those interference and obtain proper shapes of both quetiapine and IS in our experiments. Besides of this, there were some published reports about eletrokinetic suitable for FESI [23]. Therefore, electrokinetic injection mode was chosen as the optimum.

**Figure 3:**
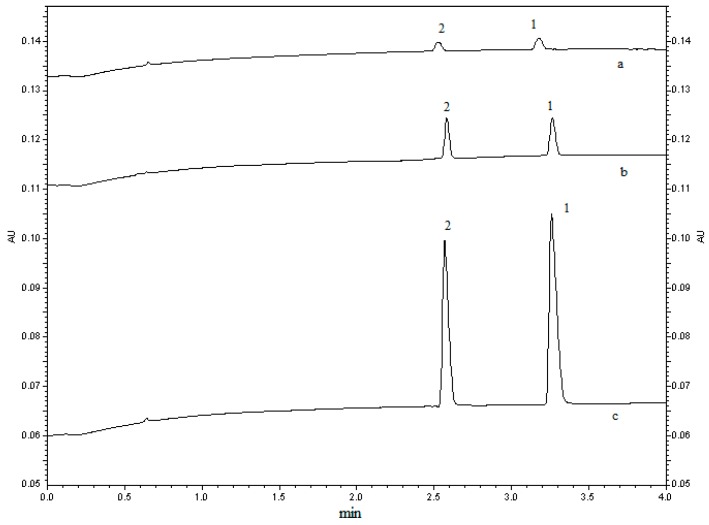
The effect of different sample solvents on FESI of quetiapine fumarate (1000 ng/mL) standard and IS (1000 ng/mL). Running buffer, 50 mM phosphate (pH 2.5); applied voltage, 13 kV (+)→(−); electrokinetic injection, 5 kV × 10 s. Electropherograms: (a) 10% buffer; (b) distilled water; (c) 65% ACN-distilled water. Peak 1: quetiapine fumarate; peak 2: IS.

FESI was usually employed as a simple and efficient technique to enhance the sensitivity in capillary zone electrophoresis [19,24,25]. In this study, the compositions of sample solvent (10% buffer, distilled water, ACN-distilled water) were investigated. As was shown in Figure 3, ACN-distilled water could be used as sample solvent to improve sensitivity. Then, the ratios of ACN to distilled water (55%, 60%, 65%, 70%, 75%) were studied to obtain a higher response of quetiapine and IS. It was found that the peak of quetiapine decreased and the current would drop to zero when the ratio of ACN greater than 70%. In the end, 65% ACN distilled water was chosen as sample solvent.

#### 2.1.3. Pre-Treatment of Sample

In this study, two methods of extracting plasma samples were compared. Firstly, protein precipitation, which was very simple and fast, was often used to process plasma samples. However, it was not suitable for quetiapine extraction from plasma due to lower sensitivity and more interference of endogenous components in beagle dog plasma. Then, the liquid-liquid extraction was explored. Increasing the sample volume before extraction and decreasing the volume of redissolve solvent can make the quetiapine and IS concentrated with no interference of endogenous components. In addition, the LLE 96-well deep format plate was used to increase sample throughput and decrease the sample preparation time effectively. Therefore, the liquid-liquid extraction in 96-well deep format plate was chosen as the pre-treatment method.

### 2.2. Method Validation

Analytical method validation was carried out according to the recommendations published by the FDA [26].

#### 2.2.1. Specificity

**Figure 4:**
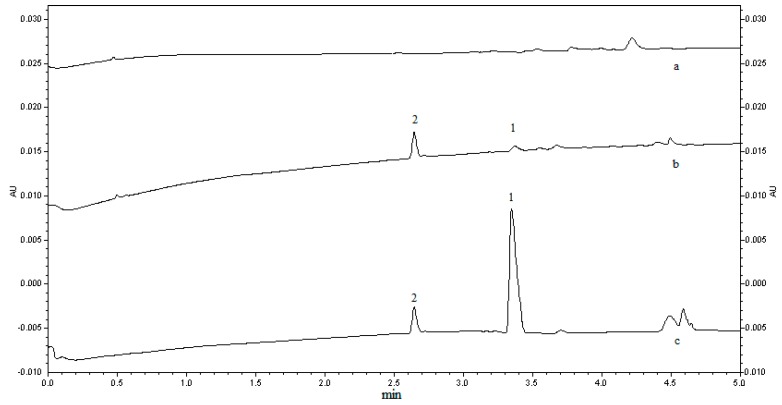
Typical chromatograms of blank plasma (a); blank plasma spiked with 1 ng/mL (LLOQ) quetiapine fumarate and 100 ng/mL IS (b); test plasma spiked with 100 ng/mL IS at 1.5 h after the dose of 200 mg quetiapine fumarate immediate release tablet (c). Peak 1: quetiapine fumarate; peak 2: IS.

Specificity of the method was investigated by blank plasma, plasma spiked with quetiapine of 1 ng/mL, and a real beagle dog plasma sample to distinguish the analytes from all potentially interfering substances. Peak purity was evaluated by means of the P/ACE System MDQ Software. The total peak purity values of quetiapine fumarate and IS were 1.0000. There were no interfering peaks from the endogenous substances observed at migration times of about 3.4 min for quetiapine and about 2.6 min for the IS, which were analyzed under the same optimized condition. Figure 4 showed the typical electropherograms for blank plasma, plasma spiked with quetiapine at 1 ng/mL (LLOQ) and a test plasma sample.

#### 2.2.2. Linearity of Calibration Curves and Lower Limit of Quantification (LLOQ)

The linearity of the CZE method was evaluated by analysis of seven concentration samples of quetiapine (1, 2, 10, 50, 200, 500 and 1000 ng/mL), each sample had a concentration of 100 ng/mL of IS. The calibration equation was y = 0.02636 × −0.002533, r^2^ = 0.9968. In this equation, y represents the peak area ratios of the analyte to the IS and x represents the plasma concentration of analyte in ng/mL. The calibration curve was achieved with a 1/x weighing factor. The LLOQ of quetiapine in beagle dog plasma was found to be 1 ng/mL with accuracy of 95.7% and precision of 12.7% (*n* = 5).

#### 2.2.3. Accuracy, Precision and Extraction Recovery

Five replicate samples at each QC concentrations (LQC, MQC, and HQC, 2, 50, 800 ng/mL) were analyzed in three separate runs. Accuracy was determined by calculating the ratios of the predicted values to the spiked concentrations and the precision was assessed by the relative standard deviations (RSD, %). The results in Table 1 show that the within- and between-day variances at three QC levels were all below 10.1%. It is shown that the accuracy was from 98.2% to 107.6%. Recovery from beagle dog plasma samples was evaluated in quintuplicate for three levels of quetiapine, the response for each concentration being compared with that from the corresponding standard solution. The extraction recovery values were shown in Table 2.

**Table 1:** Within- and between-run accuracy and precision in spiked plasma samples.

Sample Level	Low QC	Medium QC	High QC
	2 ng/mL	50 ng/mL	800 ng/mL
Within-run accuracy and precision			
Validation run 1			
Mean ± SD (ng/mL)	2.12 ± 0.13	49.63 ± 3.10	804.65 ± 43.08
Accuracy ± SD (%)	107.6 ± 6.7	101.0 ± 6.3	102.3 ± 5.5
RSD (%)	6.2	6.3	5.4
N	5	5	5
Validation run 2			
Mean ± SD (ng/mL)	1.94 ± 0.20	50.38 ± 4.55	812.36 ± 52.30
Accuracy ± SD (%)	98.2 ± 9.9	102.5 ± 9.3	103.3 ± 6.7
RSD (%)	10.1	9.0	6.4
N	5	5	5
Validation run 3			
Mean ± SD (ng/mL)	2.10 ± 0.15	51.09 ± 3.34	825.25 ± 39.85
Accuracy ± SD (%)	106.9 ± 7.5	104.0 ± 6.8	104.9 ± 5.1
RSD (%)	7.0	6.5	4.8
N	5	5	5
Between-run accuracy and precision			
Mean ± SD (ng/mL)	2.05 ± 0.17	50.36 ± 3.50	814.09 ± 42.93
Accuracy ± SD (%)	104.2 ± 8.7	102.5 ± 7.1	103.5 ± 5.5
RSD (%)	8.4	6.9	5.3
N	15	15	15

**Table 2:** Recovery values of quetiapine fumarate and IS in spiked plasma samples.

Compound	Concentration	Recovery (%)	RSD (%)
	(ng/mL)	(mean ± SD)	
Quetiapine (*n* = 5)	2	79.5 ± 9.1	11.4
	50	84.4 ± 7.3	8.6
	800	89.9 ± 4.9	5.5
IS (*n* = 15)	100	85.6 ± 7.6	8.9

#### 2.2.4. Stability

The stability of quetiapine in beagle dog plasma was evaluated by analyzing three QC levels in quintuple including short-term temperature stability, long-term stability, autosampler stability and freeze-thaw cycles stability. The stability was evaluated by the mean values and standard deviations of the ratios between the concentration found and initial concentration. The results in Table 3 showed that quetiapine fumarate had an acceptable stability at room temperature for 2 h, at −20 °C for 1 month, in the autosampler at room temperature for 8 h after liquid-liquid extraction and after three freeze-thaw cycles with the values of 94.2%–106.3%, 96.3%–105.0%, 98.1%–104.4% and 101.6%–105.7%, respectively, at the three studied concentrations. All results of stability tests indicated that the stability of quetiapine over all steps of determination was well good, so the method was proved to be applicable for routine analyses.

**Table 3:** Stability results of quetiapine fumarate in spiked plasma samples (*n* = 5).

Sample Condition	Nominal Concentration (ng/mL)	Measured Concentration (ng/mL) (Mean ± SD)	Accuracy (%)	RSD (%)
Freeze-thaw stability ^a^	2	2.04 ± 0.16	103.7	7.7
	50	49.94 ± 1.25	101.6	2.5
	800	831.55 ± 21.89	105.7	2.6
30-day stability ^b^	2	1.89 ± 0.17	96.3	8.9
	50	51.26 ± 2.54	104.3	5.0
	800	825.86 ± 39.96	105.0	4.8
Bench top stability ^c^	2	1.85 ± 0.14	94.2	7.3
	50	51.04 ± 2.68	103.8	5.3
	800	835.51 ± 29.61	106.3	3.5
Autosampler stability ^d^	2	1.93 ± 0.21	98.1	11.1
	50	51.30 ± 2.65	104.4	5.2
	800	782.29 ± 35.95	99.5	4.6

^a^: After three freeze-thaw cycles; ^b^: Stored at −20 °C; ^c^: Exposed at ambient temperature(25 °C) for 2 h; ^d^: Kept at ambient temperature(25 °C) for 8 h.

### 2.3. Application to Preclinical Pharmacokinetic Study

**Figure 5:**
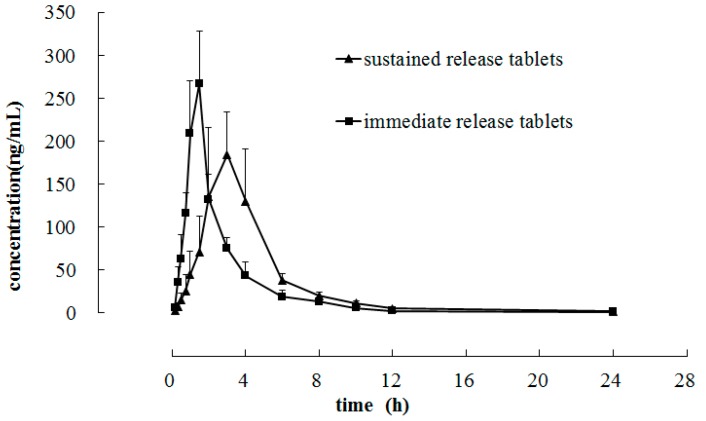
Mean plasma concentration-time curve in six beagle dogs after a single oral dose of 200 mg of quetiapine fumarate immediate release tablets and sustained release tablets.

The developed and validated FESI-CZE method was used to analyze pharmacokinetic profile of quetiapine fumarate in six beagle dogs, which received a single oral dose of 200 mg of quetiapine fumarate immediate release tablets and sustained release tablets. The sustained release tablet was test formulation. Figure 5 represented mean plasma concentration profile of quetiapine *vs.* time. The pharmacokinetic parameters were analyzed by non-compartmental method using the Bioavailability Program Package (BAPP, China Pharmaceutical University, China). After a single dose administration of quetiapine fumarate immediate release tablets, the *t*_1/2_, MRT, C_max_, *T*_max_, AUC_0–24_ and AUC_0-∞_ were (2.50 ± 1.21) h, (2.98 ± 0.43) h, (291.33 ± 15.19) ng/mL, (1.42 ± 0.20) h, (585.39 ± 110.23) ng·h/mL and (592.26 ± 109.81) ng·h/mL, respectively. For compound sustained release tablets, the same pharmacoknetic parameters were (5.42 ± 1.18) h, (5.18 ± 0.42) h, (226.07 ± 21.38) ng/mL, (3.00 ± 0.63) h, (733.46 ± 82.81) ng·h/mL and (750.30 ± 89.67) ng·h/mL, respectively.

## 3. Experimental Section

### 3.1. Chemicals and Reagents

Quetiapine fumarate standard reference material (99.9% purity) was purchased from National Institutes for Food and Drug Control (Beijing, China). The immediate release tablets formulation of quetiapine fumarate (200 mg, lot: KA187) were kindly supplied by AstraZeneca S. p. A. (Basiglio, Milan, Italy). The sustained release tablets formulation of quetiapine fumarate (200 mg, lot: C10125A) also obtained from AstraZeneca S. p. A. Mirtazapine (99.9% purity) was used as the internal standard (IS, Figure 1B) obtained from Dalian Melon Biological Technology Co. Ltd. (Dalian, China). NaH_2_PO_4_ was obtained from China Medicine (Group) Shanghai Chemical Reagent Corporation (Shanghai, PR China). Phosphoric acid was from Sigma (St. Louis, MO, USA). Methyl alcohol and acetonitrile were from MERK KGaA (Darmstadt, Germany). Methyl tert-butyl ether (chromatographic grade) was from J.T. Baker (Phillipsburg, NJ, USA). All chemicals were analytical grade or better. Beagle dog plasma (sodium heparin as an anticoagulant) was obtained from Shanghai Xingang experiment animal field. Water was deionized and purified by using a Milli-Q system (Millipore, Milford, MA, USA) and was used to prepare all aqueous solutions.

### 3.2. CE Instrumentation

Analyses were performed on a CE system consisted of a Beckman P/ACE MDQ instrument (Beckman Coulter, Brea, CA, USA) equipped with a photodiode array detection detector (PDA) and P/ACE System MDQ Software. The system of 32-Karat software (Beckman) was used for data acquisition, processing, and analysis. Detection was performed on-column at 210 nm, where quetiapine had the maximum absorption. An uncoated fused-silica capillary (31.2 cm × 75 μm i.d., effective length 21 cm) was acquired from Hebei Yongnian Optical Fiber Factory (Hebei, China).

### 3.3. Capillary Electrophoretic Conditions

The phosphate buffer (50 mM, pH 2.5) was used in this study as the background electrolyte (BGE). It was prepared by accurately weighing 0.78 g of sodium dihydrogen phosphate and thoroughly mixed it with 100 mL deionized water. Then, the pH of this buffer was adjusted to 2.5 with phosphoric acid. BGE was prepared freshly every day and filtered through a 0.45 μm hydrophilic cellulose membrane filter and degassed by sonication prior to use. The capillary temperature of the optimal separation was maintained at 25 °C by immersion of the capillary in the cartridge around of circulating cooling liquid. The separation voltage was set at 13 kV with the current of about 98 μA. The sample was injected by electrokinetic injection modes (5 kV × 10 s). A new capillary was conditioned by rinsed with 1 M NaOH for 30 min, and then flushed with 0.1 M NaOH, H_2_O, 0.1 M HCl and H_2_O (15 min each), sequentially. At the beginning of each working day the capillary was rinsed with H_2_O (2 min), 0.1 M NaOH (10 min), H_2_O (2 min), and running buffer (10 min) in regular sequence. At routine condition between runs, the capillary was rinsed in turn with 0.1 M NaOH, 0.1 M HCl, H_2_O and then BGE, each for 2 min.

### 3.4. Preparation of Stock Solutions, Calibration Samples and Quality Control Samples

Stock solutions of quetiapine fumarate and the IS were prepared in methanol at concentration of 1 mg/mL, respectively. The quetiapine fumarate working standard solutions were prepared daily by diluting the stock solution with distilled water to desired concentration of the range from 0.01 to 10 μg/mL. The stock solution of IS was diluted with distilled water to working solution (1 μg/mL). All described solutions were stored at 4 °C.

Calibration samples were obtained by diluting standard working solutions (10 μL) with drug-free beagle dog control plasma (90 μL), to span a calibration standard range of 1–1000 ng/mL (1, 2, 10, 50, 200, 500, and 1000 ng/mL). Quality control (QC) samples (2, 50, 800 ng/mL) were independently prepared by spiking appropriate amount of the working standard solutions in drug-free beagle dog control plasma.

### 3.5. Extraction Procedure

Samples were prepared by LLE in 96-well deep format plate (2 mL, Corning Life Sciences-Axygen Inc, CA USA) to increase throughput. We used an automatic multichannel electronic pipette (INTEGRA Bioscience AG, Switzerland) to complete liquid transfer steps. Plasma samples were thawed at room temperature after taken out from −80 °C freezer. 100 μL aliquot of plasma samples were added into 96-well deep format plate. Aliquots of 10 μL IS working solution (1 μg/mL) were added and vortexed homogenized for 30 s. The extraction consisted in addition of 500 μL methyl tert-butyl ether. Then the mixture was vortex-mixed for 3 min in a platform and centrifuged at 3000 *g* for 10 min. 400 μL of the supernatant organic layer was transferred from the original sample plate into a new 96-well deep format plate. And then the plate was evaporated under a gentle nitrogen flow at 30 °C. All dry residues were reconstituted by addition of 20 μL acetonitrile–water (65:35, *v*/*v*). Finally, the plate was vortex-mixed for 5 min, then centrifuged for 10 min at 4000 *g*. The supernatant was injected into the CZE system.

### 3.6. Pharmacokinetic Study in Beagle Dogs

This method was applied to determine plasma concentrations of quetiapine fumarate from a preclinical trial in beagle dogs. Beagle dogs were respectively received a single oral dose of 200 mg of quetiapine fumarate immediate release tablet and sustained release tablet. About 1 mL of blood samples were collected in heparinized centrifuge tubes at before administration (0 min) and at the time of 10, 20, 30, 45 min, and 1, 1.5, 2, 3, 4, 6, 8, 10, 12, 24 h after dosing and centrifuged at 1370 *g* for 10 min to separate the plasma fraction. The obtained plasma samples were stored at −20 °C until analysis. The study was approved by a local ethics committee.

## 4. Conclusions

A highly sensitive and effective FESI-CZE method was developed and validated for the determination of quetiapine fumarate in beagle dog plasma, with a sample pretreatment by liquid-liquid extraction in 96-well deep format plate. Under the optimal conditions, this stacking CZE method with electrokinetic injection in combine with dissolving the sample in 65% ACN yields provided a sensitivity enhancement of about 40–50 folds in comparison with the same sample dissolved in 10% buffer. The preclinical analysis process and pharmacokinetic parameters confirmed that the CZE method can be an alternative method for routine analysis of quetiapine fumarate in plasma.
